# Species Identification of Sea Turtles in Thailand Using DNA Barcoding Coupled With High‐Resolution Melting (Bar‐HRM) Analysis

**DOI:** 10.1155/sci5/6737420

**Published:** 2026-10-11

**Authors:** Anocha Poommouang, Kittisak Santisuk, Kongkiat Kittiwatanawong, Patcharaporn Kaewmong, Chaleamchat Somgird, Korakot Nganvongpanit, Kittisak Buddhachat

**Affiliations:** ^1^ Department of Biology Faculty of Science Naresuan University Phitsanulok 65000 Thailand nu.ac.th; ^2^ Phuket Marine Biological Center Phuket 83000 Thailand; ^3^ The School of Veterinary Medicine Faculty of Veterinary Medicine Chiang Mai University Chiang Mai 50200 Thailand cmu.ac.th

**Keywords:** Bar-HRM, DNA barcoding, sea turtle, species identification, wildlife forensics

## Abstract

The identification of sea turtle species relies on morphological characteristics, presenting challenges in incomplete or degraded specimens, with DNA barcoding now increasingly recognized for its potential in expediting species identification across various organisms. This study proposed a combination of DNA barcoding with high‐resolution melting (Bar‐HRM) analysis for accurate identification of sea turtles at the species level. Mitochondrial DNA (D‐loop, *cytb*, and *cox1*) and nuclear DNA (*Ahr*, *Bdnf*, *Bmp2*, *C-mos*, *Hmgb2*, *Ngf*, and *Pax1p1*) were employed for Bar‐HRM to differentiate the five sea turtle species (green turtle, *Chelonia mydas*; hawksbill, *Eretmochelys imbricata*; leatherback, *Dermochelys coriacea*; loggerhead, *Caretta caretta*; and olive ridley, *Lepidochelys olivacea*) commonly found in Thai waters and some tortoise species used as outgroup. Results demonstrated that Bar‐HRM using TurtleDloop2 and TurtleCytb2(R1) primers produced distinct normalized melting curves for all five species (100%) that their Tm value ranged 77°C–81°C and 81°C–83°C, respectively. The performance of the established approach was evaluated by a blind test of 30 samples, randomly selected for species identification by Bar‐HRM using both primers, with accuracy rates of 100%. The minimum DNA concentrations required to discriminate sea turtle and tortoise species using this method were 0.001 and 0.01 ng per reaction for TurtleDloop2 and TurtleCytb2(R1), respectively. Our findings suggested that Bar‐HRM using TurtleDloop2 and TurtleCytb2(R1) was a promising tool for the identification of sea turtle species, contributing to effective conservation endeavors and precise evaluations of their endangered status, thereby enhancing the efficiency of conservation planning initiatives.

## 1. Introduction

Seven sea turtle species are currently globally recognized as flatback (*Natator depressus*), green (*Chelonia mydas*), hawksbill (*Eretmochelys imbricata*), Kemp’s ridley (*Lepidochelys kempii*), leatherback (*Dermochelys coriacea*), loggerhead (*Caretta caretta*), and olive ridley (*Lepidochelys olivacea*) [1]. Of these, five species (*C. mydas*, *E. imbricata*, *D. coriacea*, *C. caretta*, and *L. olivacea*) are found in Thai waters [2], serving as an important habitat for sea turtle biodiversity. Sea turtles are classified as rare marine animals, and they are protected under the Wildlife Preservation and Protection Act, B.E. 2562 (2019) [3]. The five species found in Thai waters are included on the International Union for Conservation of Nature (IUCN) Red List as threatened, with a high risk of extinction. This status underscores the urgent need for conservation measures to ensure their survival [4]. Between 2017 and 2020, 610 sea turtles were recorded as stranded in the Gulf of Thailand, and this number is increasing every year. In 2021, 506 sea turtle strandings were documented, with 62% found deceased or as carcasses, while 38% were still alive [5]. This study also incorporated three tortoise species as outgroup references: the elongated tortoise (*Indotestudo elongata*), Indian star tortoise (*Geochelone elegans*), and Sulcata tortoise (*Centrochelys sulcata*). These species are commonly maintained in captivity and the pet trade in Thailand, but they are listed on the IUCN Red List due to threats, such as habitat loss and illegal hunting or trade. *I. elongata* is classified as endangered, while *C. sulcata* and *G. elegans* are categorized as vulnerable [4].

The morphological analysis of stranded sea turtle carcasses in the field is based on shell pattern [6], residual organs, meat, and skeletal remains and often poses significant challenges due to advanced degradation and loss of critical morphological features [7]. As a result, the development and application of accurate and reliable species identification tools are essential for forensic investigations [8]. Techniques used in forensic animal identification include the examination of hair, tissue, bone, morphological traits, and dental characteristics [9], which require specialized expertise [10, 11]. To improve species identification accuracy, modern approaches incorporate genetic methods, such as random amplification of polymorphic DNA (RAPD), microsatellites [12, 13], and DNA barcoding using mitochondrial DNA (mtDNA) [14, 15] and nuclear DNA (nuDNA) [16].

DNA barcoding is a highly effective method for species identification that employs short sequences of genetic material from specific regions of mtDNA, such as the displacement loop (D‐loop) [17], cytochrome b (*cytb*) [18], and cytochrome c oxidase subunit I (*cox1*) [19], and nuDNA, including genes, such as aryl hydrocarbon receptor (*Ahr*), brain‐derived neurotrophic factor (*Bdnf*), bone morphogenetic protein 2 (*Bmp2*), paired box 1 pseudogene 1 (*Pax1p1*), RNA fingerprint protein 35 (*R35*), and recombination activating gene 1 (*Rag1*) [20–23]. This technique has been successfully applied to detect cases of species trading, particularly involving rare marine animals, in contexts, such as food products and stranded carcasses [9, 12, 14, 24–27]. Although DNA barcoding provides accurate species identification, conventional sequencing–based approaches may still require additional time and cost compared with Bar‐HRM analysis. High‐resolution melting (HRM) analysis is a simple and effective technique for supporting species identification by genotyping species‐specific sequences within candidate DNA barcodes and systematically detecting unidentified genetic variations [28–32]. HRM enables the discrimination of specimens through distinctive alterations in generated melting curves, which are readily visualized [33]. This technique enables real‐time measurements by analyzing outcomes at different melting temperatures, thereby minimizing potential subjective errors associated with human biases [34–38]. DNA barcoding combined with high‐resolution melting analysis, referred to as Bar‐HRM, has been successfully employed to identify various animal species, including gecko [39], buffalo [29], and other species, such as cattle, chicken, turkey, common quail, horse, pig, rabbit, sheep, giraffe, goat, and hare [30, 31]. This approach also minimizes contamination risks and reduces the requirement for post‐PCR handling procedures [40].

This study established an efficient tool for differentiation of sea turtles in Thailand based on DNA barcodes derived from mtDNA barcodes (D‐loop, *cytb*, *cox1*) and nuDNA (*Ahr*, *Bdnf*, *Bmp2*, proto‐oncogene serine/threonine‐protein kinase mos (*C-mos*), high‐mobility group box 2 (*Hmgb2*), nerve growth factor (*Ngf*), and paired box 1 pseudogene 1 (*Pax1p1*)) coupled with HRM, so‐called Bar‐HRM. This technique was used to facilitate accurate differentiation of five sea turtle species based on differences in their HRM melting profile patterns, for better understanding population structures and improving conservation strategies.

## 2. Materials and Methods

### 2.1. Sample Collection and DNA Extraction

This study utilized samples from five sea turtle species collected from the Andaman Sea, Thailand, including green (*C. mydas*) (*n* = 3), hawksbill (*E. imbricata*) (*n* = 3), leatherback (*D. coriacea*) (*n* = 3), loggerhead (*C*. *caretta*) (*n* = 3), and olive ridley (*L*. *olivacea*) (*n* = 3), with three tortoise species included as outgroups: elongated (*I. elongata*) (*n* = 3), Sulcata (*C*. *sulcata*) (*n* = 3), and Indian star (*G*. *elegans*) (*n* = 3) which were pet obtained from owners. In addition, there were 30 sea turtle specimens which used for blind validation test of Bar‐HRM assay. The sea turtle specimens were provided by the Phuket Marine Biological Center (PMBC), Phuket, Thailand, while the tortoise specimens were obtained from the Faculty of Veterinary Medicine, Chiang Mai University, Chiang Mai, Thailand. This study was approved by the Laboratory Animal Research Center, Chiang Mai University, Thailand (Approval No. R13/2021), and all procedures followed the relevant guidelines and regulations. The authenticity and reliability of the samples were verified by experts from their respective sources. No live animal experimentation was conducted in this study, as all samples were obtained from existing specimen collections maintained by the respective institutions. Fifty‐four samples of turtles and tortoises were isolated for genomic DNA using the DNeasy Blood & Tissue Kit (QIAGEN, Germany). The DNA concentration and purity were measured using a Beckman Coulter DU 730 spectrophotometer (Beckman Coulter, USA), and the extracted DNA was diluted as 5 ng/μL stored at −20°C for further use.

### 2.2. Primer Design and Selection for Bar‐HRM

Sixteen primer sets were developed for DNA barcoding based on mtDNA regions, including the D‐loop, *cytb*, and *cox1*, as well as nuDNA regions, including *Ahr*, *Bdnf*, *Bmp2*, *C-mos*, *Hmgb2*, *Ngf*, *R35*, and *Pax1p1* from five sea turtle species and three tortoise species (Table 1). To identify suitable primers for Bar‐HRM analysis, mtDNA regions (D‐loop, *cytb*, and *cox1*) and nuDNA regions (*Ahr*, *Bdnf*, *Bmp2*, *C-mos*, *Hmgb2*, *Ngf*, and *Pax1p1*) from these eight species were retrieved from GenBank (Supporting File 1‐2). Multiple sequence alignments were performed using MultAlin (http://multalin.toulouse.inra.fr/multalin/) [41], and nucleotide diversity (window size = 20 bp) was calculated using DnaSP v5.10.01 [42]. This analysis facilitated the design of primer sets suitable for Bar‐HRM species classification. The primers produced amplicons no longer than 300 bp. Primer binding sites were in conserved regions with low nucleotide diversity (low π values), while the amplified sequences from these primer pairs exhibited sufficient variability (high π values) for species differentiation (Supporting File 1‐2).

**TABLE 1:** Oligonucleotide primers for Bar‐HRM based on DNA mtDNA and nuDNA barcodes.

Barcode	Primer name	Primer sequence (5′‐3′)	Size (bp)	Annealing temperature (°C)
D‐loop	TurtleDloop1_F	TTT RCC RYT AGC ATA TVA YYA GTA	228	50
	TurtleDloop1_R	ACA AGG RTT GCT TAT TTC TCG TGA		
	TurtleDloop2_F	TCA CGA GAA ATA AGC AAY CCT TGT	159	50
	TurtleDloop2_R	AGG CCT TTT AAA RAG RAA YGA ATG		

*cytb*	TurtleCytb1_F	GGA ATY TTY YTA GCA ATA CAY TAY T	211	50
	TurtleCytb1_R1	TRA TTC CDG TRT TTC AGG TTT CTT T		
	TurtleCytb1_R2	CTC ARA ATG ATA TTT GNC CTC ATG G	283^∗^	
	TurtleCytb2_F	CAA TGA ATC TGA GGR GGM TTY TCA	199	60
	TurtleCytb2_R1	CTT TRT AKG AGA AGT AKG GGT GGA A		
	TurtleCytb2_R2	TGA AGT TGT CTG GGT CTC C	286^∗∗^	

*cox1*	TurtleCox1 (1F)	TAT TAA TYC GYG CAG AAC TAA GCC A	262	50
	TurtleCox1 (1R)	GCR CCT GCT TCA ATT CCT GAT GA		
	TurtleCox1 (2F)	TCA TCA GGA ATT GAA GCA GGY GC	209	60
	TurtleCox1 (2R)	AAT ARK GGT GTT TGG TAT TGT GAT AT		
	TurtleCox1 (3F)	ATA TCA CAA TAC CAA ACA CCM YTA TT	191	50
	TurtleCox1 (3R)	CCA AAG AAT CAG AAT AGG TGT TG		

*Ahr*	T_AHR_F	ATC WYT GTC CTC CYG CTT CAG	247	54
	T_AHR_R	CCC AGA CTT CTC ATG ATG TTG		

*Bdnf*	T_BDNF_F	AAG TGT CAG AGG ACT AGG CAG	211	60
	T_BDNF_R	CAT AAC CCG AGA YGT GTA CAA		

*Bmp2*	T_BMP2_F	CCA AGC RAA ACA CAA ACA GCG	225	60
	T_BMP2_R	GGG ATT TTG GAA TTC ACG GAA TT		

*C-mos*	T_Cmos_F	TTT GGG TCY GTC TAC AAG GC	262	Not determined
	T_Cmos_R	GCA GGC AGC YAG TGC CAT AG		

*Hmgb2*	T_HMGB2_F	GAG AAA TAT GAA AAG GTA CA	238	Not determined
	T_ HMGB2_R	ACA TTK KTG TGT TTY CCT ACA		

*Ngf*	T_NGF_F	GCA TAC AGG CAG CAC CAA AG	238	60
	T_ NGF_R	CTG AAC AGA ACC CGA GGG GA		

*Pax1p1*	T_PAX1P1_F	CYT GAC AGA TTA CTT YRG ATK CCC C	247	60
	T_ PAX1P1_R	GAA TAT ACT TCA AAC AAG CCA C		

*Note:* The International Union of Pure and Applied Chemistry (IUPAC) defines the degenerate nucleotide codes as A (Adenine), G (Guanine), C (Cytosine), T (Thymine), D (A or G or T), K (G or T), M (A or C), N (A or C or G or T), R (A or G), W (A or T), and Y (C or T).

^∗^an asterisk indicated that the product size was obtained using the TurtleCytb1_F and TurtleCytb1_R2 primers.

^∗∗^while a double asterisk denoted successful amplification with the TurtleCytb2_F and TurtleCytb2_R2 primers.

The designed primers (Table 1) were evaluated through polymerase chain reaction (PCR) amplification using random samples from three species (*L. olivacea*, *D. coriacea*, and *C. sulcata*). Each PCR was performed in 20 μL reaction volumes consisting of 1X HOT FIREPol Blend Master Mix with 2.5 mM MgCl_2_ (Solis BioDyne, Estonia), 0.4 μM of each forward and reverse primer, 2 μL of 5 ng/μL DNA template, and distilled deionized (dd)H_2_O to complete the volume. The PCR protocol was carried out under a MiniAmp Plus Thermal cycler (Applied Biosystems, USA) including an initial denaturation at 95°C for 12 min, followed by 35 cycles of denaturation at 95°C for 30 s, annealing at a temperature gradient ranging from 50°C to 60°C for 45 s (one temperature per reaction), and an extension at 72°C for 1 min. A final extension step was conducted at 72°C for 10 min. The PCR products were assessed by gel electrophoresis on a 2% agarose gel at 120 V for 30 min.

### 2.3. Bar‐HRM Analysis

HRM analysis was conducted using a CFX Duet Real‐Time PCR System (Bio‐Rad, USA) to assess the specificity of the designed primers for differentiating eight species of sea turtles and tortoises. The reaction mixture for Bar‐HRM was performed in a total volume of 20 μL comprising 4 μL of 5X of HOT FIREPol EvaGreen HRM Mix (Solis BioDyne, Estonia), 0.4 μL of 10 μM forward primer, and 0.4 μL of 10 μM reverse primer (0.2 μM final concentration for each primer), and 2 μL of 5 ng/μL DNA. PCR protocol using an initial activation temperature at 95°C for 12 min was used, followed by 35 cycles of denaturation at 95°C for 30 s, annealing at either 50°C, 54°C or 60°C for 45 s (depending on each primer), extension at 72°C for 30 s, and a final extension at 72°C for 10 min. Before the HRM step, the DNA products were denatured at 95°C for 30 s to activate DNA association from double‐stranded DNA to single‐stranded DNA for ensuring to ensure complete denaturation and improve the reproducibility of the protocol. In the HRM step, fluorescence data (acquisition by scan time) were collected every 0.1°C/cycle with ramp rate at 5°C/second in a temperature range of 65°C–95°C. EvaGreen fluorescence dye was used to acquire melting temperature (*T*_*m*_) values and melting profiles including normalization curves and difference curves to monitor the accumulation of the amplified products during the PCR and HRM processes using the Bio‐Rad CFX Maestro V.2.3 program. The melting profiles of the eight species of sea turtles and tortoises were analyzed using Precision Melt Analysis Software to generate normalized melting curves, difference plots, and melting temperature peaks for species differentiation. Pre‐ and postmelt normalization regions were defined following HRM analysis to create normalized melting curves and different plots, ensuring an optimal temperature range for distinguishing the sea turtle and tortoise species. The DNA amplicons of different species exhibiting difference in the normalized melting curves and different plots were selected to use as DNA marker for species differentiation.

### 2.4. Sensitivity Determination of Bar‐HRM Using TurtleDloop2 and TurtleCytb2(R1) Primers

Based on the species discrimination performance and normalized melting curve profiles, Bar‐HRM using TurtleDloop2 and TurtleCytb2(R1) provided high discriminatory performance and distinct melting curve profiles among the five sea turtle species. Thereby, they were selected as candidate DNA markers to facilitating species differentiation of five sea turtle species. The sensitivity determination and validation of Bar‐HRM using these primer sets were performed. Bar‐HRM using TurtleDloop2 and TurtleCytb2(R1) primers was conducted to determine the minimum DNA concentration required to produce melting profile patterns for the five sea turtle species found stranded in Thailand: *C. mydas*, *E. imbricata*, *D. coriacea*, *C. caretta*, and *L. olivacea*. DNA templates with an initial concentration of 5 ng per reaction were serially diluted 10‐fold to obtain final concentrations of 0.0000005, 0.000005, 0.00005, 0.0005, 0.005, 0.05, 0.5, and 5 ng/μL. For DNA amplification, 2 μL of each dilution was added to the reaction mixture, resulting in final DNA amounts of 0.000001, 0.00001, 0.0001, 0.001, 0.01, 0.1, 1, and 10 ng per reaction. Distilled water was used as a negative control. The Bar‐HRM products at each concentration for each species were analyzed using 2% agarose gel electrophoresis stained with ethidium bromide (EtBr) and visualized under a UV transilluminator.

### 2.5. Validation of Bar‐HRM Using TurtleDloop2 and TurtleCytb2(R1) Primers for Species Identification

A blind validation test was used to evaluate the efficiency of Bar‐HRM using either TurtleDloop2 or TurtleCytb2(R1) primers. Five sea turtles were included in the Bar‐HRM assay as reference samples to aid species identification. Thirty samples of known specimens from PMBC, Phuket, Thailand, were randomly selected that these samples had never been used before to avoid overfitting (Supporting File 3). The samples were confirmed for species identification based on a very distinct sea turtle shell pattern by experts from PMBC, Phuket, Thailand. The normalization plot of Bar‐HRM using either TurtleDloop2 or TurtleCytb2(R1) primers was created to compare the melting curve between reference samples and testing samples for species identification.

### 2.6. Statistical Analysis

The melting temperature (*T*_*m*_) values generated from Bar‐HRM analysis using 14 primer sets across eight species were statistically analyzed to evaluate species discrimination. Differences in Tm values among species within each primer set were assessed using one‐way analysis of variance (ANOVA), followed by Tukey’s pairwise comparison test at a significance level of *p* < 0.05. Species pairs with *p*‐values lower than 0.05 were considered distinguishable based on their Tm values. The discrimination accuracy of each primer set is calculated as

(1) accuracy %=number of significantly different species pairstotal pairwise comparisons×100.

In addition, intraspecies and interspecies coefficients of variation (CV) were analyzed to evaluate primer suitability for Tm‐based species identification using Bar‐HRM. For intraspecies CV, the Tm values within each species were used to calculate CV values, and the CVs from all species were then averaged for each primer set. For interspecies CV, the mean Tm value of each species was used to calculate the standard deviation (SD) among species, followed by

(2) interspecies CV %=SDbetween speciesoverall mean Tm×100.

The discrimination ratio is calculated as follows:

(3) discrimination ratio=interspecies CVintraspecies CV.

A higher discrimination ratio indicated greater suitability of the primer set for species identification using Bar‐HRM analysis.

## 3. Results

### 3.1. Primer Design and Optimization for Species Differentiation by Bar‐HRM

In the present study, a total of 16 primer sets were designed based on three genes from mtDNA and eight genes from nuDNA. As a result, DNA amplification by PCR using the TurtleDloop1, TurtleDloop2, TurtleCytb1(R1), TurtleCytb1(R2), TurtleCox1(1), and TurtleCox1(3) primers successfully amplified DNA from all samples of the tested species at an annealing temperature of 50°C (Supporting File 4). PCR using T_AHR primers showed positive amplification for all species at 54°C, while PCR using TurtleCytb2(R1), TurtleCytb2(R2), TurtleCox1(2), T_BDNF, T_BMP2, T_NGF, and T_PAX1P1 primers was effective annealing temperature at 60°C. However, PCR using T_Cmos and T_HMGB2 primers failed to amplify DNA from any of the three tortoise species (Supporting File 4). These fourteen primer sets were used for screening Bar‐HRM to differentiate samples of the five turtles and three tortoise species (Table 1).

### 3.2. Bar‐HRM for Species Differentiation

Bar‐HRM assays using 14 selected primer sets, including TurtleDloop1, TurtleDloop2, TurtleCytb1(R1), TurtleCytb1(R2), TurtleCytb2(R1), TurtleCytb2(R2), TurtleCox1(1), TurtleCox1(2), TurtleCox1(3), T_AHR, T_BDNF, T_BMP2, T_NGF, and T_PAX1P1, successfully amplified DNA from all tested species (Figure 1a–n). The normalized melting curves generated by different primer sets varied among species, demonstrating the potential of Bar‐HRM for discriminating all five sea turtle species and three tortoise species as reference for nontarget species specificity test (Figure 1).

**FIGURE 1:**
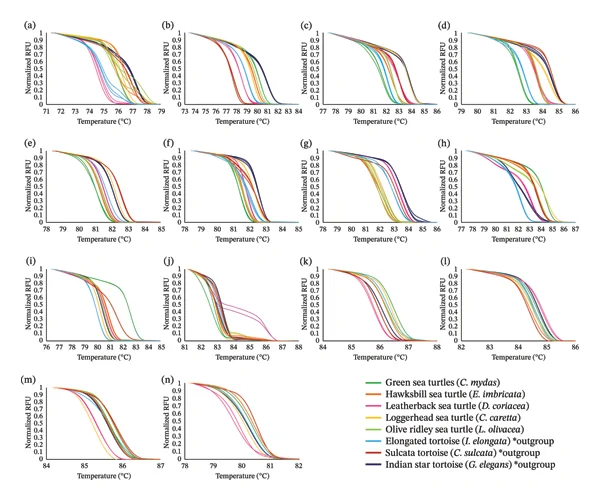
Normalized melting curves of Bar‐HRM using each of the fourteen primer sets to distinguish between five turtle and three tortoise species. The primer sets were used in this study including TurtleDloop1 (a), TurtleDloop2 (b), TurtleCytb1(R1) (c), TurtleCytb1(R2) (d), TurtleCytb2(R1) (e), TurtleCytb2(R2) (f), TurtleCox1(1) (g), TurtleCox1(2) (h), TurtleCox1(3) (i), T_AHR (j), T_BDNF (k), T_BMP2 (l), T_NGF (m), and T_PAX1P1 (n).

Analysis of melting temperature (Tm) values revealed both small and large variations among species depending on the primer set used (Figure 2a). For example, loggerhead turtles showed a broad Tm range when analyzed using the TurtleCytb1(R1) primer, whereas leatherback, hawksbill, and green turtles displayed narrower Tm ranges that overlapped with the loggerhead turtle Tm range (Figure 2a). To evaluate primer suitability, intraspecies and interspecies CV were used to calculate the discrimination ratio (Supporting File 5). Among all primer sets, Bar‐HRM using TurtleDloop2 showed the highest discrimination ratio due to its low intraspecies CV combined with high interspecies CV, indicating strong species discrimination ability. This was followed by T_AHR, T_BDNF, and TurtleCytb2(R1) primers, respectively (Figure 2b).

FIGURE 2The melting temperature derived from Bar‐HRM for species differentiation of five turtle and three tortoise species. The Tm obtained from Bar‐HRM using 14 primer sets including TurtleDloop1, TurtleDloop2, TurtleCytb1(R1), TurtleCytb1(R2), TurtleCytb2(R1), TurtleCytb2(R2), TurtleCox1(1), TurtleCox1(2), TurtleCox1(3), T_AHR, T_BDNF, T_BMP2, T_NGF, and T_PAX1P1. (a) The average Tm of each species with 95% confidential interval (CI). (b) Discrimination ratio derived from the intraspecies coefficient variation (CV) and interspecies CV was used to select the primer suitability for species differentiation. (c) Tukey’s pairwise comparison and discrimination accuracy of each primer for aiding species differentiation. Raw data are shown in Supporting File 5.

**Figure:**
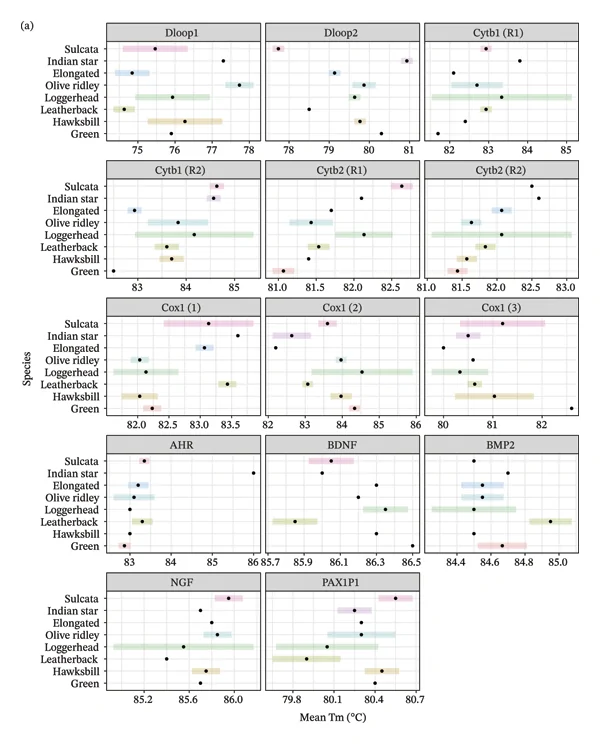

**Figure:**
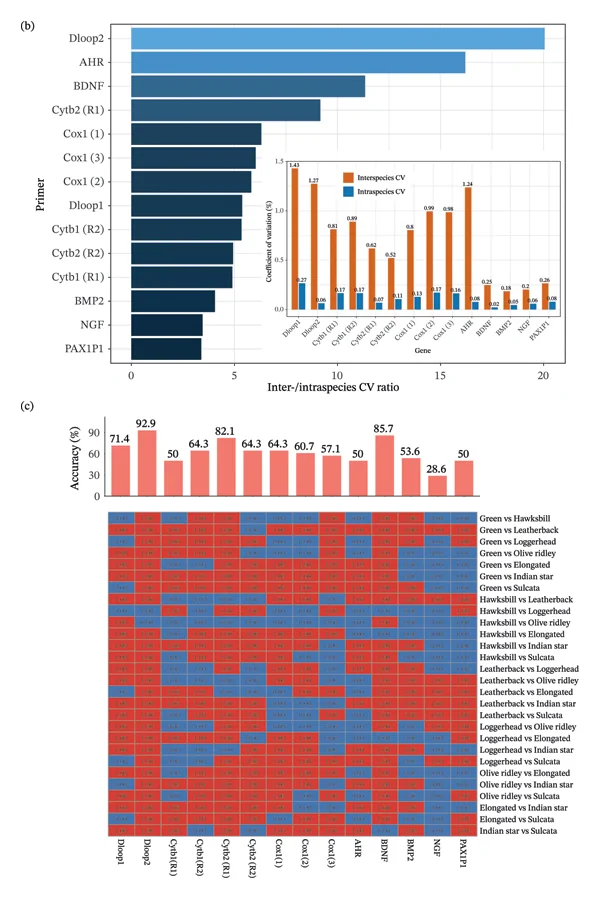

Tukey’s pairwise comparison was further used to assess the performance of Tm‐based species differentiation. Consistent with the CV analysis, Bar‐HRM using TurtleDloop2 provided the highest discrimination performance, successfully separating 20 species pairs with a discrimination accuracy of 92.9%. This was followed by T_BDNF (85.7%) and TurtleCytb2(R1) (82.1%) (Figure 2c).

Although Tm values from these primer sets were not sufficient to completely differentiate all five turtle and three tortoise species, analysis of the normalized melting curve profiles enabled clearer species classification, highlighting the superior discriminatory power of melting curve shape. Among the 14 primer sets evaluated, TurtleDloop2, T_BDNF, and TurtleCytb2(R1) showed the best performance for species discrimination based on Tm analysis (Figure 2c). However, the melting curves generated using T_BDNF were relatively similar among species (Figure 1k), whereas TurtleCytb2(R1) produced more distinct melting curve profiles for each species. Importantly, both primer sets successfully amplified DNA from all tested species.

When focusing specifically on sea turtles based on the normalized melting curve profiles, Bar‐HRM assays using the TurtleCytb1(R1) and T_PAX1P1 primers were able to clearly distinguish all five sea turtle species. In addition, Bar‐HRM assays using the nuDNA primers T_AHR, T_BDNF, T_BMP2, T_NGF, and T_PAX1P1 successfully differentiated leatherback turtles (*D. coriacea*) from the other sea turtle species based on their normalized melting curves.

### 3.3. Sensitivity Determination of Bar‐HRM Using TurtleDloop2 or TurtleCytb2(R1) Primers

The detection sensitivity of Bar‐HRM using TurtleDloop2 and TurtleCytb2(R1) primers was evaluated for the sea turtle species *C. mydas*, *E. imbricata*, *D. coriacea*, *C. caretta*, and *L. olivacea* (Figure 3a–j). DNA samples were successfully detected within the concentration range 0.000001–10 ng/μL, with the lowest DNA amount leading to primer dimer formation. This is evident from the shift in the melting peak observed at lower concentrations compared with higher concentrations (Supporting File 6). Bar‐HRM using TurtleDloop2 (Figure 3a, c, e, g, and i) exhibited a detection limit of 0.001 ng per reaction for all five sea turtle species. By contrast, Bar‐HRM using TurtleCytb2(R1) (Figure 3b, d, f, h, and j) successfully amplified DNA at a minimum concentration of 0.01 ng per reaction for all species.

FIGURE 3Sensitivity of Bar‐HRM using TurtleDloop2 and TurtleCytb2(R1) primers for the five species of sea turtles. Amplification plots and agarose gel electrophoresis in TurtleDloop2 (a, c, e, g, i) and TurtleCytb2(R1) (b, d, f, h, j) of *C. mydas* (a, b), *E. imbricata* (c, d), *D. coriacea* (e, f), *C. caretta* (g, h), and *L. olivacea* (i, j). The DNA of each species was used in different amounts, ranging from 0.000001 to 10 ng per reaction.

**Figure:**
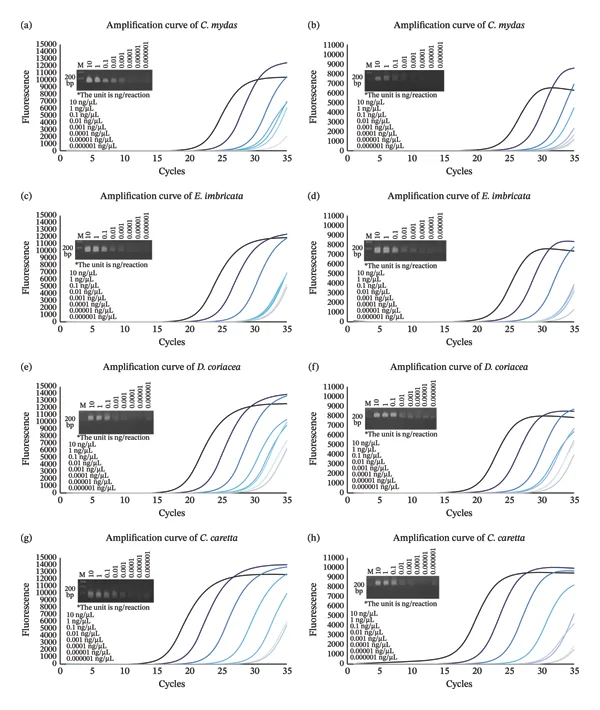

**Figure:**
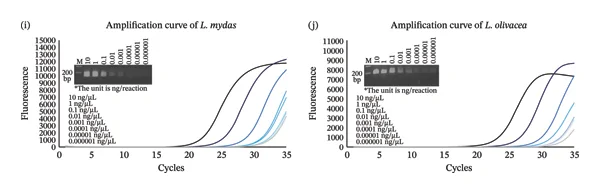

### 3.4. Validation of Bar‐HRM Using TurtleDloop2 or TurtleCytb2(R1) Primers

The accuracy of species identification using Bar‐HRM was assessed through a blind test (Figure 4a,b). The results demonstrated that Bar‐HRM using the TurtleDloop2 (Figure 4a) and TurtleCytb2(R1) (Figure 4b) primers produced normalized melting curves that closely matched the reference samples from reference species. This consistency was observed across all 30 blind samples, achieving a 100% identification success rate (Supporting File 3).

**FIGURE 4:**
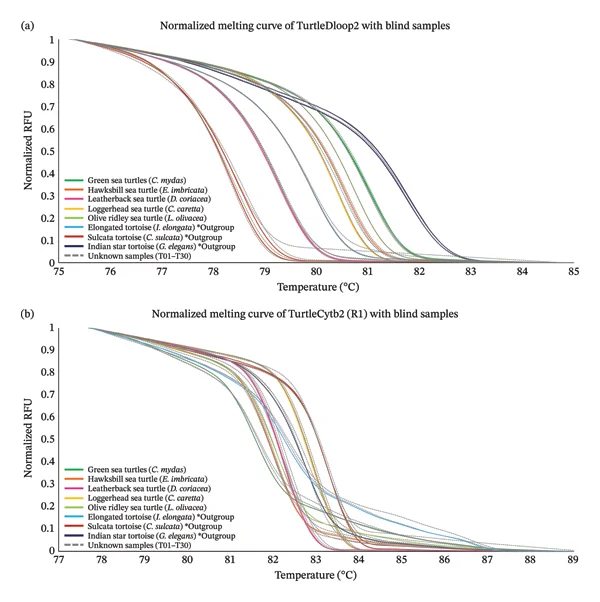
Validation process for identifying 30 blind samples from five sea turtle species by analyzing normalized melting curves using Bar‐HRM. Each graph presents the blind sample test results obtained with Bar‐HRM using TurtleDloop2 (a) and TurtleCytb2(R1) (b). The normalized melting curves in all charts closely matched known species, indicating a high degree of similarity between the blind samples and the reference species.

## 4. Discussion

There is an urgent need for reliable identification methods to support sea turtle conservation, with five threatened species found in Thai waters. Traditional morphological approaches are limited due to carcass degradation, prompting the use of molecular techniques. This study developed an efficient Bar‐HRM assay using mitochondrial (D‐loop, *cytb*, and *cox1*) and nuclear (*Ahr*, *Bdnf*, *Bmp2*, *C-mos*, *Hmgb2*, *Ngf*, and *Pax1p1*) DNA markers. The Bar‐HRM method provided a rapid, accurate, and cost‐effective tool for differentiating sea turtle species, thereby supporting potential forensic and conservation applications, population monitoring, and conservation planning. Bar‐HRM provided rapid, accurate, and cost‐effective identification of the five sea turtle species in Thai waters in single reaction. Bar‐HRM using two primer sets, TurtleDloop2 and TurtleCytb2(R1), achieved 100% species differentiation based on distinct normalized melting curves. This approach resolved a major challenge in marine conservation forensics by overcoming the limitations of morphological and sequencing methods, particularly for degraded samples from strandings and illegal trade.

All the primer sets were designed based on multiple alignments of sequences from sea turtles and tortoises, with the tortoises serving as an outgroup to test the discriminatory power of the assay through normalized melting curve analysis. Among the primers assessed, TurtleDloop2, TurtleCytb2(R1), TurtleCox1(3), and T_PAX1P1 successfully identified the five sea turtle species. For optimal Bar‐HRM performance, the primers should produce normalized melting curves that clearly distinguish sea turtles from the outgroup without overlap. This requirement is critical to ensure the specificity of the assay in complex forensic or ecological samples where the origins of the species are unknown. In this study, Bar‐HRM using either TurtleDloop2 or TurtleCytb2(R1) primers demonstrated outstanding efficiency in differentiating between the five sea turtle species. The findings demonstrated the reliability and effectiveness of Bar‐HRM in accurately distinguishing sea turtle species, reinforcing its potential as a robust molecular tool for species identification in conservation and ecological studies. The Bar‐HRM method demonstrated significant advantages over traditional and alternative genetic techniques for species identification. Morphological identification of stranded carcasses is often impossible due to advanced decomposition [7], while conventional DNA barcoding via Sanger sequencing, though highly accurate, is time‐consuming (typically requiring 24–48 h for results) and costly US$10–15 per sample for reagents and sequencing. Therefore, DNA barcoding is not suitable for high‐throughput screening or rapid forensic response [43]. Bar‐HRM is a closed‐tube method that eliminates post‐PCR processing, thereby reducing the risk of contamination and providing results within not exceeding 3 h at a significantly lower cost (US$2–4 per sample). Other PCR‐based methods, such as PCR‐RFLP, involve additional enzymatic digestion steps and gel electrophoresis, which are more labor‐intensive (adding 2–4 h of hands‐on time) and less amenable to automation [44]. In this study, Bar‐HRM achieved 100% accuracy in the blind test, demonstrating its potential as a reliable and rapid tool for routine monitoring and emergency stranding response.

DNA barcodes, the D‐loop and *cytb* regions, were identified as the most effective molecular markers for identifying sea turtles, with DNA sequences from mtDNA more suitable for species identification than those from nuDNA due to the higher mutation rates of the former [45, 46]. The faster evolution of mtDNA leads to greater variability in specific regions, such as the D‐loop, cytochrome b (*cytb*), and cytochrome c oxidase subunit I (*cox1*) [47, 48]. Mitochondrial (mt)DNA is highly sensitive to species‐level differences, particularly among closely related species [46, 49]. However, mtDNA is also maternally inherited without recombination, providing a stable lineage for tracing evolutionary relationships and accurate species identification. By contrast, nuDNA undergoes recombination, which can obscure lineage‐specific signals [50, 51]. These properties establish mtDNA markers as highly effective for species differentiation, and they have been widely and successfully applied for genetic diversity, population genetics, and phylogenetic classification across diverse animal taxa, including killer whales [52], dugongs [15], fish [53], and various turtle and tortoise species [48, 54, 55]. With respect, the use of only the DNA marker from mtDNA is limited for hybrid detection which might occur in the reproduction of two species of sea turtles. Therefore, nuDNA is necessitated for hybrid detection as well. So far, six hybrid combinations among five sea turtle species—olive ridleys, Kemp’s ridleys, loggerheads, hawksbills, and green turtles—have been reported in different parts of the world, including Canada, Brazil, Japan, and Australia [56].

The conserved regions in mtDNA are ideal for designing universal primers, enabling amplification across diverse species, which is especially critical in marine biodiversity studies [57]. The mtDNA exists in multiple copies per cell (hundreds to thousands), dissimilar to nuDNA, which is present as a single copy per chromosome per cell. This abundance makes mtDNA easier to extract and amplify, even from degraded or low‐quality samples [46, 51]. This feature is particularly advantageous in identifying stranded or decomposed sea turtle species and stems from fundamental differences in degradation kinetics between the two genomes: nuDNA typically becomes unamplifiable within days to weeks in decomposing soft tissue, whereas mtDNA persists for much longer. Experimental studies across taxa support this distinction. In degraded mouse tissues, mtDNA remained amplifiable for longer periods than nuDNA across various temperatures [58], and in human dental tissues, mtDNA degraded more slowly, allowing successful amplification even in highly compromised samples [59]. Research on human decomposition in soil demonstrated that mtDNA remained detectable and amplifiable for extended periods, even under harsh environmental conditions [60]. The mtDNA and nuDNA barcodes used in this study clearly differentiated the leatherback sea turtles from other sea turtle species because it belongs to a different evolutionary lineage. Leatherback turtles are classified in the family Dermochelyidae, whereas the other sea turtle species belong to the family Cheloniidae [61]. This clear phylogenetic separation underscores how Bar‐HRM reflects deep evolutionary divergences. The leatherback’s unique morphological and physiological adaptations, such as its soft shell and gigantothermy, are mirrored in its distinct genetic signature, which was reliably captured [62]. Consequently, when a hybrid is suspected, whether based on morphological anomalies or genetic discrepancies, the Bar‐HRM method with appropriate nuDNA markers provides a robust tool for definitive confirmation.

Although mtDNA markers demonstrated greater efficacy in distinguishing between sea turtle species, nuDNA markers retained significant value for hybrid analysis due to their biparental inheritance and recombination. These properties allowed nuDNA markers to capture genetic contributions from both parents, which mtDNA could not achieve. A study in the South Western Atlantic, using a combination of mtDNA and nuDNA markers, revealed immature marine turtle hybrids that would have been misidentified using mtDNA haplotypes alone [56], providing valuable insights into hybrid detection using nuDNA markers. Bar‐HRM with nuDNA markers showed potential for different species, such as *D. coriacea* (general nuDNA), *C. mydas* (T_AHR, T_PAX1P1), *C. mydas* and *C. caretta* (T_BDNF), *E. imbricata* (T_BMP2, T_PAX1P1), and *C. caretta* (T_NGF). In sea turtles, recorded hybridization events, particularly among *E*. *imbricata*, *C*. *caretta*, and *L*. *olivacea*, rely on nuclear loci for the reliable detection of both F1 and backcrossed hybrids [63]. This strategy has also been applied in mammals, with nuclear SNPs or microsatellites used to identify hybrid individuals and introgression into *Canis* species [64, 65] and Felidae species [66, 67]. Bar‐HRM is not equally effective for hybrid detection across all species pairs. The method shows clear limitations in distinguishing hybrids between closely related Cheloniidae species, such as *C. caretta* (loggerhead) and *E. imbricata* (hawksbill), due to their highly similar DNA sequences. Thus, Bar‐HRM using nuDNA primers alone is unsuitable for all cases. Due to deep evolutionary divergence, a hybrid between *D. coriacea* and any Cheloniidae species would likely produce a distinct, unambiguous melting profile, making it detectable. However, hybrids among closely related Cheloniidae species (e.g., *C. caretta* × *E. imbricata*) cannot be reliably distinguished because their normalized melting curves overlap. Therefore, for closely related taxa, Bar‐HRM alone lacks the resolution for confident hybrid detection, and a multilocus nuclear genotyping approach is essential.

The advantages of mtDNA were reflected in the high sensitivity of the Bar‐HRM method. Specifically, Bar‐HRM using the TurtleDloop2 primer generated distinct melting profiles with only 0.001 ng of DNA per reaction, displaying the high sensitivity of the assay. When analyzing highly degraded samples from stranding events or wildlife forensic cases, the DNA is often fragmented and of low quantity. The robustness of the mtDNA‐based Bar‐HRM assay ensures reliable species identification even when the sample quality is poor, a common limitation that obstructs the use of nuDNA markers. However, degraded forensic specimens and processed products were not directly evaluated in this study. Therefore, additional validation using naturally degraded tissues and commercially processed samples is still required before routine forensic implementation. The performance of mtDNA‐based Bar‐HRM is highly competitive, as evidenced by a comparable detection threshold of 0.001 ng/μL achieved in processed food products [68], and validates the principle of using mtDNA for reliable identification of degraded samples, as previously demonstrated in forensic science [69].

Bar‐HRM serves as a rapid, accurate tool for sea turtle identification, a critical first step in monitoring marine animal strandings [70]. Integrating species data with stranding frequency, environmental conditions, and pollution levels is vital for assessing risks of population decline and extinction [71, 72]. The practical implications for conservation are significant. Implementing Bar‐HRM in stranding networks and forensic laboratories can enhance data quality on mortality causes and threats, informing targeted conservation actions, such as bycatch mitigation [73]. Furthermore, the ability of Bar‐HRM to identify species from processed items or tissue remnants provides a powerful asset for combating the illegal wildlife trade by enabling law enforcement to prosecute cases involving protected species [8]. A key limitation of this study is that the Bar‐HRM primers were designed and validated using only the five sea turtle species found in Thai waters. Therefore, the applicability of the assay to the two other sea turtle species, the flatback (*Natator depressus*) and Kemp’s ridley (*Lepidochelys kempii*), remains uncertain. Although these two species were not included in the experimental validation, their reference sequences from public databases were compared with those of the five species analyzed in this study. Using the TurtleDloop2 and TurtleCytb2(R1) primers, normalized melting curves were simulated with the uMelt program [74]. The results indicated that both additional species could potentially be distinguished, particularly with TurtleDloop2 (data not shown). These findings suggest that the primers developed in this study may have the potential to discriminate among all seven globally recognized sea turtle species. However, this in silico prediction requires further validation using biological samples of *N. depressus* and *L. kempii*. Moreover, genetic variation among sea turtle populations from different geographic regions may influence the melting curve profiles and, consequently, the ability of the assay to reliably distinguish these species. Nevertheless, a blind test incorporating the two untested species is recommended for future validation. Without validation in these taxa, the method cannot be considered universally robust for sea turtle identification worldwide, while relying exclusively on regional genetic datasets may overlook phylogeographic variations across populations, which have been shown to influence DNA melting profiles and species resolution [75]. This limitation underscores the need for broader testing using samples from diverse geographic ranges to ensure global transferability of the assay. Expanding the reference library with comprehensive mtDNA and nuDNA sequences from all seven sea turtle species would enhance global applicability and allow the detection of hybrids and population‐level variation, thereby strengthening the method’s utility in conservation genetics and wildlife forensics.

The Bar‐HRM assay developed in this study provides a rapid and reliable tool for species‐level identification of sea turtles, supporting population surveys and conservation programs. Effective management should also integrate ecological and anthropogenic factors, including stranding locations, environmental conditions, food availability, and fisheries impacts. For species with small populations and high extinction risk, targeted measures, such as rehabilitation programs, antitrafficking enforcement, ecosystem monitoring, and public engagement initiatives, are essential. Spatial planning must further ensure the long‐term suitability of sea turtle habitats. Future research should extend primer validation across broader geographic ranges to capture potential phylogeographic variations [75] and incorporate the flatback (*Natator depressus*) and Kemp’s ridley (*Lepidochelys kempii*) to establish a global assay and evaluate multilocus Bar‐HRM approaches that multiplex mtDNA and nuDNA markers. Integrating machine learning for automated curve classification could further standardize interpretation and enable high‐throughput monitoring [38].

## Author Contributions

Kittisak Buddhachat and Korakot Nganvongpanit conceptualized the idea of this study. Kittisak Buddhachat administered the project funding. Kittisak Buddhachat, Korakot Nganvongpanit, and Anocha Poommouang designed the experiments. Anocha Poommouang carried out all the experiments, analyzed the data, created visualizations, and performed statistical analyses. Kittisak Santisuk contributed to conducting certain experiments and assisted in data analysis. Kongkiat Kittiwatanawong and Patcharaporn Kaewmong provided the specimens. Chaleamchat Somgird identified the species of tortoises based on morphological characteristics. Kittisak Buddhachat and Anocha Poommouang collaborated in writing the manuscript.

## Funding

The study was funded by the Faculty of Science, Naresuan University, Thailand, R2569E026; the Global and Frontier Research University Fund, Naresuan University, Thailand, R2566C051.

## Disclosure

All the authors reviewed and approved the final version of the manuscript for publication.

## Conflicts of Interest

The authors declare no conflicts of interest.

## Supporting Information

Additional supporting information can be found online in the Supporting Information section.

## Supporting information


**Supporting Information 1** File Name: Supporting File 1. Title: Development and evaluation of primer Sets for mtDNA barcoding. Description: This file provides the sequences and detailed characteristics of the primer sets developed for the amplification of mitochondrial DNA (mtDNA) barcoding regions. The aimed regions include the displacement loop (D‐loop), cytochrome b (cytb), and cytochrome c oxidase subunit I (cox1).

**Supporting Information 2** File Name: Supporting File 2. Title: Development and evaluation of primer Sets for nuDNA barcoding. Description: This file contains the sequences and detailed characteristics of the primer sets developed for the amplification of nuclear DNA (nuDNA) regions. The targeted genes include Aryl Hydrocarbon Receptor (Ahr), Brain‐Derived Neurotrophic Factor (Bdnf), Bone Morphogenetic Protein 2 (Bmp2), Proto‐Oncogene Serine/Threonine‐Protein Kinase Mos (C‐mos), High Mobility Group Box 2 (Hmgb2), Nerve Growth Factor (Ngf), and Paired Box 1 Pseudogene 1 (Pax1p1). These primers were designed to complement the mtDNA markers for comprehensive genetic analysis.

**Supporting Information 3** File Name: Supporting File 3. Title. The blind samples for validation of Bar‐HRM and its results. Description: This file presents the sea turtle samples used for blind validation test of Bar‐HRM and its results.

**Supporting Information 4** File Name: Supporting File 4. Title. Gel Electrophoresis validation of primer specificity. Description: This file presents the gel electrophoresis results from the initial PCR screening of the designed primer sets. The images confirm the specificity and amplification success of each primer pair, validating their performance and ensuring the absence of non‐specific products or primer‐dimer artifacts before their application in the subsequent Bar‐HRM analysis.

**Supporting Information 5** File Name: Supporting File 5. Title. The value of melting temperature of each primer from Bar‐HRM and Description: The file presents the raw data of melting temperature (Tm) derived from Bar‐HRM using 14 primer sets and the information from the statistical analysis to determine the suitable primer for species differentiation of sea turtles.

**Supporting Information 6** File Name: Supporting File 6. Title. The melting peak of different DNA concentrations from Bar‐HRM of five sea turtle species. Description: The file presents the melting peak obtained from the sensitivity determination of five turtle species in different concentration of DNA target used in HRM reaction.

## Data Availability

The multiple alignments of all DNA barcodes from mitochondria and nuDNA and their nucleotide diversity for primer design are deposited in Supporting File 1 and Supporting File 2, respectively. The sea turtle samples used for blind validation test of Bar‐HRM and its results are provided in Supporting File 3. The raw data of agarose gel electrophoresis of PCR optimization of each primer designed in this study are displayed in Supporting File 4. The raw data of melting temperature (Tm) derived from Bar‐HRM using 14 primer sets and the information from the statistical analysis are depicted in Supporting File 5. The melting peak obtained from the sensitivity determination of five turtle species in different concentrations of DNA target used in the HRM reaction are shown in Supporting File 6.

## References

[1] Mazaris A. D. , Schofield G. , Gkazinou C. , Almpanidou V. , and Hays G. C. , Global Sea Turtle Conservation Successes, Science Advances. (2017) 3, no. 9, 10.1126/sciadv.1600730. PMC5606703 28948215

[2] Chomchat P. , Kaewmong P. , Sirinarumitr K. , Sera K. , Noda J. , and Sirinarumitr T. , Study of Trace Elements in Stranded Green Turtles (*Chelonia Mydas*), Hawksbill Turtles (*Eretmochelys Imbricata*), and Olive Ridley Turtles (*Lepidochelys olivacea*) in Gulf of Thailand and Andaman Sea, Journal of Veterinary Medical Science. (2023) 85, no. 5, 557–564, 10.1292/jvms.22-0348. PMC10209466 36948643

[3] Royal Thai Government Gazette , Wildlife Preservation and Protection Act B.E. 2562, 2019, Royal Thai Government Gazette, https://www.ratchakitcha.soc.go.th/DATA/PDF/2562/A/082/T_0001.PDF.

[4] International Union for Conservation of Nature and Natural Resources (IUCN) , The IUCN Red List of Threatened Species, 2023, https://www.iucnredlist.org/.

[5] Prampramote J. , Boonhoh W. , Intongead S. et al., Association of Ocean Macroplastic Debris With Stranded Sea Turtles in the Central Gulf of Thailand, Endangered Species Research. (2022) 47, 333–343, 10.3354/esr01182.

[6] da Paixão W. R. , Paixão T. M. , da Costa M. C. , Andrade J. O. , Pereira F. G. , and Komati K. S. , Texture Classification of Sea Turtle Shell Based on Color Features: Color Histograms and Chromaticity Moments, International Journal of Artificial Intelligence and Applications. (2018) 9, no. 2, 55–67.

[7] Work T. M. and Balazs G. H. , Pathology and Distribution of Sea Turtles Landed as Bycatch in the Hawaii-Based North Pacific Pelagic Longline Fishery, Journal of Wildlife Diseases. (2010) 46, no. 2, 422–432, 10.7589/0090-3558-46.2.422. 20688635

[8] Ogden R. , Dawnay N. , and McEwing R. , Wildlife DNA Forensics-Bridging the Gap Between Conservation Genetics and Law Enforcement, Endangered Species Research. (2009) 9, no. 3, 179–195, 10.3354/esr00144.

[9] Martinez I. and Daníelsdóttir A. K. , Identification of Marine Mammal Species in Food Products, Journal of the Science of Food and Agriculture. (2000) 80, no. 4, 527–533.

[10] Linacre A. and Tobe S. S. , An Overview to the Investigative Approach to Species Testing in Wildlife Forensic Science, Investigative Genetics. (2011) 2, no. 1, 1–9, 10.1186/2041-2223-2-2. PMC3032691 21232099

[11] Alacs E. A. , Georges A. , FitzSimmons N. N. , and Robertson J. , DNA Detective: A Review of Molecular Approaches to Wildlife Forensics, Forensic Science, Medicine and Pathology. (2010) 6, no. 3, 180–194, 10.1007/s12024-009-9131-7. 20013321

[12] Baker C. S. , Lukoschek V. , Lavery S. et al., Incomplete Reporting of Whale, Dolphin and Porpoise ‘Bycatch’ Revealed by Molecular Monitoring of Korean Markets, Animal Conservation. (2006) 9, no. 4, 474–482, 10.1111/j.1469-1795.2006.00062.x.

[13] Poommouang A. , Piboon P. , Buddhachat K. et al., Microsatellite Polymorphism and the Population Structure of Dugongs (*Dugong dugon*) in Thailand, Animals. (2022) 12, no. 3, 10.3390/ani12030235. PMC8833502 35158560

[14] Baker C. S. , Cooke J. G. , Lavery S. et al., Estimating the Number of Whales Entering Trade Using DNA Profiling and Capture-Recapture Analysis of Market Products, Molecular Ecology. (2007) 16, no. 13, 2617–2626, 10.1111/j.1365-294x.2007.03317.x. 17594434

[15] Poommouang A. , Kriangwanich W. , Buddhachat K. et al., Genetic Diversity in a Unique Population of Dugong (*Dugong dugon*) Along the Sea Coasts of Thailand, Scientific Reports. (2021) 11, no. 1, 10.1038/s41598-021-90947-4. PMC8172547 34078973

[16] Cardinali I. , Tancredi D. , and Lancioni H. , The Revolution of Animal Genomics in Forensic Sciences, International Journal of Molecular Sciences. (2023) 24, no. 10, 10.3390/ijms24108821. PMC10218398 37240167

[17] Parkanyi V. , Ondruska L. , Vasicek D. , and Slamecka J. , Multilevel D-Loop PCR Identification of Hunting Game, Applied & Translational Genomics. (2013) 3, no. 1, 1–7, 10.1016/j.atg.2013.03.001. PMC4881763 27275406

[18] Parson W. , Pegoraro K. , Niederstätter H. , Föger M. , and Steinlechner M. , Species Identification by Means of the Cytochrome B Gene, International Journal of Legal Medicine. (2000) 114, no. 1, 23–28, 10.1007/s004140000134. 11197623

[19] Li P. , Li D. , Hong Y. et al., Combining DNA Mini-Barcoding and Species-Specific Primers PCR Technology for Identification of *Heosemys grandis* , Frontiers in Ecology and Evolution. (2022) 10, 10.3389/fevo.2022.822871.

[20] Rafael M. S. , Laizé V. , and Cancela M. L. , Identification of *Sparus aurata* Bone Morphogenetic Protein 2: Molecular Cloning, Gene Expression and in Silico Analysis of Protein Conserved Features in Vertebrates, Bone. (2006) 39, no. 6, 1373–1381, 10.1016/j.bone.2006.06.021. 16919510

[21] Reitzel A. M. , Karchner S. I. , Franks D. G. et al., Genetic Variation at Aryl Hydrocarbon Receptor (*AHR*) Loci in Populations of Atlantic Killifish (*Fundulus heteroclitus*) Inhabiting Polluted and Reference Habitats, BMC Ecology and Evolution. (2014) 14, no. 1, 10.1186/1471-2148-14-6. PMC3899389 24422594

[22] Thomson R. C. , Spinks P. Q. , and Shaffer H. B. , A Global Phylogeny of Turtles Reveals a Burst of Climate-Associated Diversification on Continental Margins, Proceedings of the National Academy of Sciences of the United States of America. (2021) 118, no. 7, 10.1073/pnas.201221511. PMC7896334 33558231

[23] Wu W. , Kong X. , Jia Y. et al., An Overview of PAX1: Expression, Function and Regulation in Development and Diseases, Frontiers in Cell and Developmental Biology. (2022) 10, 10.3389/fcell.2022.1051102. PMC9649799 36393845

[24] Sholl T. G. C. , de Moura J. F. , Ott P. H. et al., Cytochrome B Sequencing for the Species Identification of Whale Carcasses Washed Ashore in Brazil, Marine Biodiversity Records. (2013) 6, 10.1017/s1755267212001157.

[25] Sholl T. G. C. , Do Nascimento F. F. , Leoncini O. , Bonvicino C. R. , and Siciliano S. , Taxonomic Identification of Dolphin Love Charms Commercialized in the Amazonian Region Through the Analysis of Cytochrome B DNA, Journal of the Marine Biological Association of the United Kingdom. (2008) 88, no. 6, 1207–1210, 10.1017/s002531540800043x.

[26] S ilva V. , Skueresky N. , Lopes F. et al., Integrating Morphology and DNA Barcoding to Assess Cetacean Diversity in Brazil, Mammal Research. (2021) 66, no. 2, 349–369, 10.1007/s13364-021-00555-w.

[27] Teramitsu I. , Yamamoto Y. , Chiba I. et al., Identification of Novel Cytochrome P450 1A Genes From Five Marine Mammal Species, Aquatic Toxicology. (2000) 51, no. 2, 145–153, 10.1016/s0166-445x(00)00113-2. 11064121

[28] Wittwer C. T. , High Resolution DNA Melting Analysis: Advancements and Limitations, Human Mutation. (2009) 30, no. 6, 857–859, 10.1002/humu.20951. 19479960

[29] Sakaridis I. , Ganopoulos I. , Argiriou A. , and Tsaftaris A. , A Fast and Accurate Method for Controlling the Correct Labeling of Products Containing Buffalo Meat Using High Resolution Melting (HRM) Analysis, Meat Science. (2013) 94, no. 1, 84–88, 10.1016/j.meatsci.2012.12.017. 23403299

[30] Lopez-Oceja A. , Nuñez C. , Baeta M. , Gamarra D. , and De Pancorbo M. M. , Species Identification in Meat Products: A New Screening Method Based on High Resolution Melting Analysis of Cyt B Gene, Food Chemistry. (2017) 237, 701–706, 10.1016/j.foodchem.2017.06.004. 28764056

[31] Ouso D. O. , Otiende M. Y. , Jeneby M. M. et al., Three-Gene PCR and High Resolution Melting Analysis for Differentiating Vertebrate Species Mitochondrial DNA for Biodiversity Research and Complementing Forensic Surveillance, Scientific Reports. (2020) 10, no. 1, 10.1038/s41598-020-61600-3. PMC7075967 32179808

[32] Buglione M. , Petrelli S. , Notomista T. , de Filippo G. , Gregorio R. , and Fulgione D. , Who is Who? High Resolution Melting Analysis to Discern Between Hare Species Using Non-Invasive Sampling, Conservation Genetics Resources. (2020) 12, no. 4, 727–732, 10.1007/s12686-020-01153-9.

[33] Saadi-Ben Aoun Y. , Souguir H. , Chouaieb H. et al., A High Resolution Melting Analysis (HRM) PCR Assay for the Detection and Identification of Old World *Leishmania* Species, PLoS Neglected Tropical Diseases. (2024) 18, no. 12. 10.1371/journal.pntd.0012762 PMC11684767 39715284

[34] Erali M. and Wittwer C. T. , High Resolution Melting Analysis for Gene Scanning, Methods. (2010) 50, no. 4, 250–261, 10.1016/j.ymeth.2010.01.013. PMC2836412 20085814

[35] Reed G. H. , Kent J. O. , and Wittwer C. T. , High Resolution DNA Melting Analysis for Simple and Efficient Molecular Diagnostics, Pharmacogenomics. (2007) 8, no. 6, 597–608, 10.2217/14622416.8.6.597. 17559349

[36] Tulsiani S. M. , Craig S. B. , Graham G. C. et al., High Resolution Melt Curve Analysis of Random Amplified Polymorphic DNA Markers, for the Characterisation of Pathogenic *Leptospira* , Annals of Tropical Medicine and Parasitology. (2010) 104, no. 2, 151–161, 10.1179/136485910x12607012374037. 20406582

[37] Vossen R. H. , Aten E. , Roos A. , and den Dunnen J. T. , High Resolution Melting Analysis (HRMA): More Than Just Sequence Variant Screening, Human Mutation. (2009) 30, no. 6, 860–866, 10.1002/humu.21019. 19418555

[38] Wittwer C. T. , Reed G. H. , Gundry C. N. , Vandersteen J. G. , and Pryor R. J. , High Resolution Genotyping by Amplicon Melting Analysis Using Lcgreen, Clinical Chemistry. (2003) 49, no. 6, 853–860, 10.1373/49.6.853. 12765979

[39] Su Y. , Ding D. , Yao M. et al., Specific DNA Mini-Barcoding for Identification of *Gekko Gecko* and its Products, Chinese Medicine. (2020) 15, no. 1, 10.1186/s13020-020-00382-2. PMC7526243 33014122

[40] Jiang M. , Li J. , Zhou J. , Xing C. , Xu J. J. , and Guo F. , High Resolution Melting Analysis for Rapid and Sensitive MYD88 Screening in Chronic Lymphocytic Leukemia, Oncology Letters. (2019) 18, no. 1, 814–821, 10.3892/ol.2019.10342. PMC6540357 31289558

[41] Corpet F. , Multiple Sequence Alignment With Hierarchical Clustering, Nucleic Acids Research. (1988) 16, no. 22, 10881–10890, 10.1093/nar/16.22.10881. PMC338945 2849754

[42] Librado P. and Rozas J. , Dnasp v5: A Software for Comprehensive Analysis of DNA Polymorphism Data, Bioinformatics. (2009) 25, no. 11, 1451–1452, 10.1093/bioinformatics/btp187. 19346325

[43] Hebert P. D. , Cywinska A. , Ball S. L. , and DeWaard J. R. , Biological Identifications Through DNA Barcodes, Proceedings of the Royal Society B: Biological Sciences. (2003) 270, no. 1512, 313–321, 10.1098/rspb.2002.2218. PMC1691236 12614582

[44] Teletchea F. , Molecular Identification Methods of Fish Species: Reassessment and Possible Applications, Reviews in Fish Biology and Fisheries. (2009) 19, no. 3, 265–293, 10.1007/s11160-009-9107-4.

[45] Galtier N. , Nabholz B. , Glémin S. , and Hurst G. D. D. , Mitochondrial DNA as a Marker of Molecular Diversity: A Reappraisal, Molecular Ecology. (2009) 18, no. 22, 4541–4550, 10.1111/j.1365-294X.2009.04380.x. 19821901

[46] Allio R. , Donega S. , Galtier N. , and Nabholz B. , Large Variation in the Ratio of Mitochondrial to Nuclear Mutation Rate Across Animals: Implications for Genetic Diversity and the Use of Mitochondrial DNA as a Molecular Marker, Molecular Biology and Evolution. (2017) 34, no. 11, 2762–2772, 10.1093/molbev/msx197. 28981721

[47] Gissi C. , Iannelli F. , and Pesole G. , Evolution of the Mitochondrial Genome of Metazoa as Exemplified by Comparison of Congeneric Species, Heredity. (2008) 101, no. 4, 301–320, 10.1038/hdy.2008.62. 18612321

[48] Lalitha R. and Chandavar V. R. , Analysis of Genetic Diversity in CYTB and Control Region Sequences of *Melanochelys trijuga* (Schweigger, 1812) From Karnataka, Journal of Asia-Pacific Biodiversity. (2018) 11, no. 3, 346–352, 10.1016/j.japb.2018.05.001.

[49] Hellberg M. , No Variation and Low Synonymous Substitution Rates in Coral Mtdna Despite High Nuclear Variation, BMC Ecology and Evolution. (2006) 6, no. 1, 10.1186/1471-2148-6-24. PMC1431588 16542456

[50] Hagström E. , Freyer C. , Battersby B. J. , Stewart J. B. , and Larsson N. G. , No Recombination of Mtdna After Heteroplasmy for 50 Generations in the Mouse Maternal Germline, Nucleic Acids Research. (2013) 42, no. 2, 1111–1116, 10.1093/nar/gkt969. PMC3902947 24163253

[51] Ladoukakis E. D. and Zouros E. , Evolution and Inheritance of Animal Mitochondrial DNA: Rules and Exceptions, Journal of Biological Research-Thessaloniki. (2017) 24, no. 1, 10.1186/s40709-017-0060-4. PMC5282644 28164041

[52] Mitani Y. , Kita Y. F. , Saino S. , Yoshioka M. , Ohizumi H. , and Nakahara F. , Mitochondrial DNA Haplotypes of Killer Whales Around Hokkaido, Japan, Mammal Study. (2021) 46, no. 3, 205–211, 10.3106/ms2020-0072.

[53] Zhang J. and Hanner R. , Molecular Approach to the Identification of Fish in the South China Sea, PLoS One. (2012) 7, no. 2, 10.1371/journal.pone.0030621. PMC3281855 22363454

[54] Duchene S. , Frey A. , Alfaro-Núñez A. , Dutton P. H. , Gilbert M. T. P. , and Morin P. A. , Marine Turtle Mitogenome Phylogenetics and Evolution, Molecular Phylogenetics and Evolution. (2012) 65, no. 1, 241–250, 10.1016/j.ympev.2012.06.010. 22750111

[55] Komoroske L. M. , Jensen M. P. , Stewart K. R. , Shamblin B. M. , and Dutton P. H. , Advances in the Application of Genetics in Marine Turtle Biology and Conservation, Frontiers in Marine Science. (2017) 4, 10.3389/fmars.2017.00156.

[56] Brito C. , Vilaça S. T. , Lacerda A. L. et al., Combined Use of Mitochondrial and Nuclear Genetic Markers Further Reveal Immature Marine Turtle Hybrids Along the South Western Atlantic, Genetics and Molecular Biology. (2020) 43, no. 2, 1–11, 10.1590/1678-4685-GMB-2019-0098. PMC7199923 32353097

[57] Wu Q. , Nakano T. , Ishida S. et al., Development of Universal PCR Primers for the Environmental DNA Metabarcoding of Cephalopod (Mollusca) Diversity, Marine Environmental Research. (2025) 208, 10.1016/j.marenvres.2025.107094. 40245615

[58] Foran D. R. , Relative Degradation of Nuclear and Mitochondrial DNA: An Experimental Approach, Journal of Forensic Sciences. (2006) 51, no. 4, 766–770, 10.1111/j.1556-4029.2006.00176.x. 16882217

[59] Higgins D. , Rohrlach A. B. , Kaidonis J. , Townsend G. , and Austin J. J. , Differential Nuclear and Mitochondrial DNA Preservation in Post-Mortem Teeth With Implications for Forensic and Ancient DNA Studies, PLoS One. (2015) 10, no. 5, 10.1371/journal.pone.0126935. PMC4438076 25992635

[60] Emmons A. L. , DeBruyn J. M. , Mundorff A. Z. , Cobaugh K. L. , and Cabana G. S. , The Persistence of Human DNA in Soil Following Surface Decomposition, Science & Justice. (2017) 57, no. 5, 341–348, 10.1016/j.scijus.2017.05.002. 28889863

[61] Bowen B. W. and Karl S. A. , Population Genetics and Phylogeography of Sea Turtles, Molecular Ecology. (2007) 16, no. 23, 4886–4907, 10.1111/j.1365-294X.2007.03542.x. 17944856

[62] Davenport J. , Fraher J. , Fitzgerald E. et al., Ontogenetic Changes in Tracheal Structure Facilitate Deep Dives and Cold Water Foraging in Adult Leatherback Sea Turtles, Journal of Experimental Biology. (2009) 212, no. 21, 3440–3447, 10.1242/jeb.034991. 19837885

[63] Vilaça S. T. , Piccinno R. , Rota-Stabelli O. et al., Divergence and Hybridization in Sea Turtles: Inferences From Genome Data Show Evidence of Ancient Gene Flow Between Species, Molecular Ecology. (2021) 30, no. 23, 6178–6192, 10.1111/mec.16113. PMC9292604 34390061

[64] Dziech A. , Identification of Wolf-Dog Hybrids in Europe-An Overview of Genetic Studies, Frontiers in Ecology and Evolution. (2021) 9, 10.3389/fevo.2021.760160.

[65] Podbielska A. , Piórkowska K. , and Szmatoła T. , Microsatellite-Based Genetic Structure and Hybrid Detection in Alpacas Bred in Poland, Animals. (2021) 11, no. 8, 10.3390/ani11082193. PMC8388510 34438651

[66] Oliveira R. , Randi E. , Mattucci F. , Kurushima J. D. , Lyons L. A. , and Alves P. C. , Toward a Genome-Wide Approach for Detecting Hybrids: Informative SNPs to Detect Introgression Between Domestic Cats and European Wildcats (*Felis silvestris*), Heredity. (2015) 115, no. 3, 195–205, 10.1038/hdy.2015.25. PMC4814236 26103945

[67] Senn H. V. , Ghazali M. , Kaden J. et al., Distinguishing the Victim from the Threat: Snp-Based Methods Reveal the Extent of Introgressive Hybridization Between Wildcats and Domestic Cats in Scotland and Inform Future in Situ and Ex Situ Management Options for Species Restoration, Evolutionary Applications. (2019) 12, no. 3, 399–414, 10.1111/eva.12720. PMC6383845 30828363

[68] Zhao J. , Yang W. , Cai H. , Cao G. , and Li Z. , Current Progress and Future Trends of Genomics-Based Techniques for Food Adulteration Identification, Foods. (2025) 14, no. 7, 10.3390/foods14071116. PMC11989147 40238250

[69] Dawnay N. , Ogden R. , McEwing R. , Carvalho G. R. , and Thorpe R. S. , Validation of the Barcoding Gene COI for Use in Forensic Genetic Species Identification, Forensic Science International. (2007) 173, no. 1, 1–6, 10.1016/j.forsciint.2006.09.013. 17300895

[70] Koch V. , Peckham H. , Mancini A. , and Eguchi T. , Estimating at-Sea Mortality of Marine Turtles From Stranding Frequencies and Drifter Experiments, PLoS One. (2013) 8, no. 2, 10.1371/journal.pone.0056776. PMC3577704 23483880

[71] Flint J. , Flint M. , Limpus C. J. , and Mills P. C. , The Impact of Environmental Factors on Marine Turtle Stranding Rates, PLoS One. (2017) 12, no. 8, 10.1371/journal.pone.0182548. PMC5542510 28771635

[72] Chou W. R. , Wu P. Y. , and Li T. H. , Assessing the Relationship Between Sea Turtle Strandings and Anthropogenic Impacts in Taiwan, Journal of Marine Science and Engineering. (2023) 11, no. 10, 10.3390/jmse11101962.

[73] Madduppa H. H. , Bahri S. , Subhan B. et al., DNA Barcoding of Sea Turtles (*dermochelyidae* and *Cheloniidae*) and its Protocol Using Different Tissues Quality: Implication to Conservation Managers, IOP Conference Series: Earth and Environmental Science. (2019) 278, no. 1, 10.1088/1755-1315/278/1/012041.

[74] Dwight Z. , Palais R. , and Wittwer C. T. , Umelt: Prediction of High-Resolution Melting Curves and Dynamic Melting Profiles of PCR Products in a Rich Web Application, Bioinformatics. (2011) 27, no. 7, 1019–1020, 10.1093/bioinformatics/btr065. 21300699

[75] Shamblin B. M. , Bolten A. B. , Abreu-Grobois F. A. et al., Geographic Patterns of Genetic Variation in a Broadly Distributed Marine Vertebrate: New Insights Into Loggerhead Turtle Stock Structure From Expanded Mitochondrial DNA Sequences, PLoS One. (2014) 9, no. 1, 10.1371/journal.pone.0085956. PMC3900438 24465810

